# Supplementary material for: Understanding the Knowledge, Attitudes, and Practices of Healthy Eating among Adolescents in Chongqing, China: An Empirical Study Utilizing Structural Equation Modeling
Source: Nutrients. 2024 Jan 4;16(1):167. doi: 10.3390/nu16010167 (PMC10780587; doi:10.3390/nu16010167)
Supplement: Supplementary file 1 [file nutrients-16-00167-s001.zip › nutrients-2732420-supplementary.pdf]

**File S1: Questionnaire on healthy diet knowledge, attitude and behavior of middle school students in Chongqing**

**Basic information** (Tick "√" on the option you think is appropriate and fill in the specific information on "_____")

1. Your date of birth: _______ Year _______ month

2. Your gender: ① male ② female

3. Your ethnicity is: ① Han ② Miao ③ Tujia ④ Hui ⑤ Others ____

4. Your grade: ①Grade one of junior high school ②Grade two of junior high school ③Grade three of junior high school

5. Are you currently living on campus? ① Yes ② No

6. Your family's place of residence: ① Urban ② Rural

7. Whether you are the only child: ① Yes ② No, there are ____ brothers and sisters (excluding yourself)

8. Your father's education level: ① Primary school and below ② Junior middle school ③ High school/Vocational high school ④ Junior college/Bachelor degree and above ⑤ Don't know

9. Your mother's education level: ① Primary school and below ② Junior middle school ③ High school/Vocational high school ④ Junior college/Bachelor degree and above ⑤ Don't know

10. Last measured, your height:____cm, your weight:____kg

**Knowledge part(Multiple choice)**

1. The "Dietary Guidelines for Chinese Residents" put forward six core recommendations, in addition to less salt and oil, control sugar and limit alcohol; Waste elimination, which of the following does it include?

① Variety of food, mainly cereals; Eat less and move more for a healthy weight; Eat more fish, poultry, eggs, lean meat; Eat a moderate amount of vegetables, fruits, milk, soybeans.

② Various foods, fruits and vegetables, milk, soybeans; Eat dynamic balance, healthy weight; Eat more grains; Eat a moderate amount of fish, poultry, eggs, lean meat.

③Variety of food, mainly cereals; Eat dynamic balance, healthy weight; Eat more fruits and vegetables, dairy products, soybeans; Eat a moderate amount of fish, poultry, eggs, lean meat.

④Variety of food, fruits and vegetables, milk, soybeans; Eat dynamic balance, healthy weight; Eat more fish, poultry, eggs, lean meat; Eat cereals and tubers in moderation.

⑤ Don't know

2. Based on the nutritional labels of the following two dairy products, please determine which one belongs to a dairy product with higher nutritional value?

| Nutrition Facts List A | | | Nutrition Facts List B | | |
|---|---|---|---|---|---|
| Items | Per 100ml | NRV% | Items | Per 100ml | NRV% |
| Energy | 146KJ | 2% | Energy | 261KJ | 3% |
| Protein | 1.0g | 2% | Protein | 3.0g | 5% |
| Fat | 1.2g | 2% | Fat | 3.6g | 6% |
| Carbohydrate | 4.6g | 2% | Carbohydrate | 4.5g | 2% |
| Sodium | 67mg | 3% | Sodium | 60mg | 3% |
| Ingredients: water, whole milk powder, white sugar, whey protein powder, etc | | | Ingredients: raw milk | | |

3. Which of the statements about daily combinations and consumption of vegetables and fruits is incorrect?

① Vegetables should be included in the meal.

②Should eat more dark vegetables (such as green leafy vegetables, carrots, tomatoes, etc.), accounting for at least half of the total vegetables.

③You should eat fruit every day, and ensure that you eat 200g to 350g (the size of two fists) of fruit every day.

④Drinking fresh fruit juice can replace eating fruit.

⑤ Don't know

4. A salty diet is most likely to cause which diseases?

① Hypertension ② Diabetes ③ Obesity ④ Gout ⑤ Unclear

5. Long-term consumption of sugary drinks increases the risk of which of the following diseases? (Multiple options available)

① Caries ② Obesity ③ Affects blood pressure ④ Affects bone development ⑤ Don't know

6. What are the advantages of "separate dining system"?(Multiple options available)

① Reduce the risk of oral transmission of infectious diseases (such as hepatitis A).

② Help control food intake.

③ It's good for reducing food waste.

④ it's good for personalizing diets.

⑤ Don't know

**Attitudes part (Please tick " √ " in the corresponding position of your selected option)**

| Items | Strong agree | Agree | Neutral | Disagree | Strongly disagree |
|---|---|---|---|---|---|
| I think it is important to know nutrition for health. | | | | | |
| I would like to use my nutrition knowledge to guide my daily eating behavior. | | | | | |
| "Separate dining system"* or "public chopsticks and spoons" should be promoted in public restaurants. | | | | | |
| There are great food safety risks in roadside food stalls. | | | | | |

*The separate dining system refers to a dining method where there is no cross-contact or mixing of tableware, dishes, and beverages during the meal [1].

**Behavior part (Please tick "√" in the corresponding position of the option you selected)**

| Items | Always | Often | Sometimes | Occasionally | Never |
|---|---|---|---|---|---|
| I insist on three meals a day at a fixed time. | | | | | |
| Even when I'm not thirsty, I hydrate myself regularly. | | | | | |
| When I buy food, I look at the production date and expiration date. | | | | | |
| When dining in the dining hall, I will inform the staff in advance how much food I want to eat. | | | | | |


Reference:

[1].Standard, P.N. Guidance on individual dining system in catering service. Availabe online:http://c.gb688.cn/bzgk/gb/showGb?type=online&hcno=C07AF2A9821A56C245A1A6465A016787 (accessed on June 21, 2020).
